# Molecular typing of *Mycobacterium tuberculosis* complex isolated from pulmonary tuberculosis patients in central Ethiopia

**DOI:** 10.1186/s12879-017-2267-2

**Published:** 2017-03-01

**Authors:** Zufan Bedewi, Adane Worku, Yalemtsehay Mekonnen, Getnet Yimer, Girmay Medhin, Gezahegne Mamo, Rembert Pieper, Gobena Ameni

**Affiliations:** 1 Aklilu Lemma Institute of Pathobiology, Addis Ababa University, P.O. Box 1176, Addis Ababa, Ethiopia; 2 College of Natural Sciences, Microbial, Cellular and Molecular Biology Department, Addis Ababa University, P.O. Box 1176, Addis Ababa, Ethiopia; 3 College of Natural Science and Computational Science, Department of Biology, Hawassa University, P.O. Box 05, Hawassa, Ethiopia; 4 College of Veterinary Medicine and Agriculture, Addis Ababa University, P.O. Box 34, DebreZeit, Ethiopia; 5 College of Health Sciences, Addis Ababa University, P.O. Box 1176, Addis Ababa, Ethiopia; 6 J.Craig Venter Institute, 9704 Medical Center Drive, Rockville, MD USA

**Keywords:** Diversity of strain, *M. tuberculosis*, Central Ethiopia

## Abstract

**Background:**

Identification of the types of strains of *Mycobacterium tuberculosis* (*M. tuberculosis*) complex causing tuberculosis (TB) could contribute to TB control program of specific geographic region as well as it could add knowledge onto the existing literature on TB worldwide. The objective of the present study was to identify the species and strains of *M. tuberculosis* complex causing pulmonary tuberculosis in central Ethiopia.

**Methods:**

A health institution- based cross-sectional study was conducted on 338 smear positive TB cases visiting three hospitals between October 2012 and September 2013. Morning and spot sputum samples were collected before the starting of treatment regimens. Thus, a total of 338 pooled sputum samples collected from these cases. Samples were cultured on Löwenstein Jensen media and the isolates were identified by the region of difference (RD) 9 based polymerase chain reaction (PCR) and spoligotyping.

**Result:**

Of the total isolates 98.6% of the isolates were identified to be *M. tuberculosis* while the remaining 1.4% were identified as *M. africanum*. Further, typing of *M. tuberculosis* using spoligotyping lead to the identification of 90 different strains of *M. tuberculosis*. Of these strains, 32 were clustered consisting of more than one isolate while the remaining 58 strains were unique consisting of single isolate. Thus, 79.3% (223/281) of the isolates were found in the clustered while only 20.6% (58/281) of the strains were unique. Forty-five of the spolgotyping patterns were registeredin the SITVIT2 or SpolDB4 database in while the remaining 45 were notfound in the database and hence were orphan strains. The dominant strains were SIT53, SIT149, and SIT54, consisting of 43, 37 and 34 isolates, respectively. Classification of the spoligotype patterns using TB-insight RUN TB-Lineage showed that 86.8, 6.4, 5, 1.4% ofthe isolatesbelonged to the Euro-American lineage, East-African-Indian, Indo-oceanic and *M. africanum,* respectively.

**Conclusion:**

The identification of clustered and new strains using spolygotyping in present study does not give conclusive finding as spoligotyping has low discriminatory power. Thus, further identification of these isolates using mycobacterial interspersed repetitive unit-variable number tandem repeat (MIRU-VENTR) and or whole genome sequencing (WGS) recommended.

## Background

Tuberculosis (TB) is one of an infectious disease, affecting millions of people worldwide. According to the 13^th^ Annual TB Report in 2014, there were 9.6 million new TB cases and 1.5 million TB deaths annually [1]. The spread of human immunodeficiency virus (HIV) and drug-resistant TB have exacerbated the situation.

**Fig. 1:**
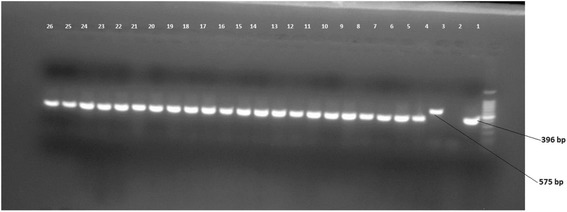
Picture of gel electrophoresis for RD9 deletion typing of *M. tuberculosis* isolates. Lane 1 is a ladder, lane 2 is *M. Tuberculosis* control, lane 3 is Negative control (molecular grade H2O), lane 4 is *M. bovis* control and lane 5–26 is culture isolate of *M. Tuberculosis*

Ethiopia has high rate of TB infection and the disease is one of major public health problems in the country [2]. According to a WHO report, the country is one among the world’s 22 countries with high TB burden [1]. The annual incidence of new TB cases was estimated to be 207/100,000 and the prevalence of TB in the country was 200/100,000 [1]. The country is one of the 27 high MDR-TB countries; ranked 15^th^ with more than 5000 estimated MDR-TB patients each year [1, 2]. MDR TB was 1.6% of new cases and 12% of re-treatment cases [1].

Effective TB control program requires understanding of its epidemiology including the strains of *M.tuberculosis*complex (MTBC) circulating in the population [3]. Molecular epidemiological studies have been used to provide valuable information onthe spread of tubercle bacilli in outbreaks [4] and also contributed to study the transmission dynamics of TB [5]. Moreover, such studies can help in distinguishing exogenous reinfection from endogenous reactivation [6]. Additionally, molecular epidemiological studiescan be used to identify laboratory cross contamination [7] and to track the geographic distribution and spread of clones, including Multi drug resistant strains [8]. Furthermore, molecular typing has shown the large differences in pathobiological properties of MTBC species [9].

In Ethiopia, TB is a major public health problem and few molecular epidemiological studies have been done so far in some part of the country. While the genetic diversity of *M. tuberculosis* lineages in the country has been investigated [10–13], there is little or no data in some part of the country including this study area. The availability of such information will help study the phylogenetic characteristics of an organism, which in turn will provide new insight into the natural history of *M. tuberculosis* and Knowledge on the *Mycobacterium tuberculosis* strains circulating in the country communities is useful for epidemiology, transmission, and is essential in the control of the disease.

Thus, identificationthe types of strains of MTBC causing TB in a specific geographic region could contribute to the strengthening the TB control program of that specific geographic region, as it can alert personnel in the TB Control Program so that they can monitor the transmission of special strains such as drug resistant and virulent strains. In addition, identificationthe types of strains circulating in a specific geographic region could help in adding additional knowledge on the existing literature on TB worldwide. To our knowledge there is scarcity of information on strains of *M. tuberculosis* of circulating in central Ethiopia. Therefore, the objective of the present study was to identify the species and strains of MTBC causing pulmonary TB in central Ethiopia.

## Methods

### Study area

This study was performed at three different sites in central Ethiopia. These sites were Woliso and Atat towns and their surroundings in the southwest of Addis Ababa at a distance of 114 km and 187 km, respectively. The third site was Fiche town and its surrounding in the northwest of Addis Ababa at 115km. Sample collection was performed at hospitals located at these three sites, namely, St. Lukas, Atat and Fitche hospitals located at Woliso, Atat and Fitche tows, respectively. Sputum samples were collected from smear positive TB cases visiting these three hospitals.

### Study design and study subjects

A health institution-based cross-sectional study was conducted on 338 smear positive TB patients visiting three hospitals, between October 2012 and September 2013. Three consecutive sputum samples (spot, early morning and spot) were collected from each of these 338 smear positive TB patient and pooled together for culturing. Sample collection was performed prior to the beginning of TB treatment. TB patients less than 18 years old excluded from this study. Socio-demographic data of the patients were obtained from the medical records of all patients.

Those samples positive for acid fast bacilli (AFB) by Ziehl-Neelsen (ZN) staining technique were collected labeled and samples from the individual patient pooled together. These sputum samples for culture were stored at −20°C and then transported in a cold box (at +4°C) to Aklilu Lemma Institute of Pathobiology (ALIPB), Addis Ababa, within a week for culture.

### Culture

Morning and spot sputum samples were collected and processed for culture following the WHO Guideline [14]. Briefly, equal volume of 4% NaOH was mixedwith sputum sample and the mixture was centrifuged at 3000 rpm for 15 min at room temperature. After decanting the supernatant the sediment was neutralized with 2 N HClusing phenol red as an indicator. Neutralization was achieved when the color of the solution was changed from purple to yellow. Thereafter, 100 μl of the suspension was inoculated ontotwo sterile LJ medium slopes (which were enriched with either pyruvate or glycerol). The inoculated media were then incubated at 37°C in slanted positionfor 1 week and upright position for 4–5 weeks. The growth of the bacteria was read every week until the 8^th^week of culture.

### Preparation of specimens for molecular typing

Colonies were removed from the surface of LJ medium and suspended in 200 μl of sterile double distilled water. Thereafter, the colonies and water were mixed thoroughly and then, the mixture was heated at 80°C for 1 h in water bath. This is followed by centrifugation after which the supernatant was collected and used for amplification [15].

### Region of difference (RD) 9-based polymerase chain reaction (PCR)

Identification of *M. tuberculosis* from the other members of *M. tuberculosis* complexspecies was done using RD9-based PCR. RD9-PCRwas performed on heat-killed cells to confirm the presence or absence of RD9 using three primers namely, RD9flankF, RD9 IntR, and RD9flankR. Amplification was done by standard thermo cycler (VWR Thermo cycler, UK). The PCR amplification mixture used consisted of 10 μlHotStarTaqMaster Mix (Qiagen, United Kingdom), 7.1μl distilled water, 0.3 μl of each three primers and 2 μl of DNA template(heat killed cells), giving a total volume of 20 μl. The PCR reaction was heatedat 95°C for 15 min after which it was subjected to 35 cycles consisting of 95 °C for one min, 55 °C for one minutes, and 72 °C for one minute. Thereafter, the reaction mixture was maintained at 72 °C for 10 min following which the product was removed from the thermocycler and run on agarose gel electrophoresis. For gel electrophoresis, 8 μl PCR products was mixed with 2 μl loading dye, loaded onto 1.5% agarose gel and electophoresed at 100 V and 500 mA for 45 min. The gel was then visualized using a computerized Multi- Image Light Cabinet (VWR). *M. tuberculosis* H37Rv, *M. bovis*bacilleCalmette-Guérin, and water were included as positive and negative controls. Interpretation of the result was based on bands of different sizes, as previously described by Parsons et al. [16].

### Spoligotyping

Isolates that were positive for *M. tuberculosis* by RD9 PCR were further characterized by spoligotyping following the procedure described by Kamerbeeket al [17] and by observing the instructions of the spoligotype kit supplier (Ocimum Biosolutions Company, Iisselstein, and the Netherlands). The direct repeat (DR) region of the isolate was amplified by PCR using oligonucleotide primers (DRa and DRb) derived from the DR sequence [17]. The amplified biotinylated products were hybridized to a set of 43 oligonucleotides covalently bound to a membrane (Animal and Plant Health Agency, Great Britain). Bound fragments were incubated with streptavidinperoxidase conjugate and hybridizing DNA was detected by the enhanced chemiluminescence method, by exposure to X-ray film (Hyperfilm ECL, Amersham) as specified by the manufacturer’s instruction. The presence and absence of spacers was visualized on the film as black and white squares, respectively. Characterized strains of *M. bovis*and *M. tuberculosis* H37Rv were used as positive controls, whereas Qiagen distilled water (Qiagen company, Germany) was used as a negative control.

### Use of SpolDB4 and Run TB-Lineage for the identification of strains and lineages

The results of spoligotyping were converted into octal and binary formats. These binary and octal formats of the strains were entered into query box so that the name of the strains are retrieved from the database if the spoligotype pattern of the strain in question fits the pattern that has already been registered in the SPolDB4 database [18] and at http://www.pasteur-guadeloupe.fr:8081/SITVITDemo/ [10]. If the pattern of the strain in question has not been registered in SPolDB prior to this study, the strain was considered as an orphan. In this study, an isolate is referred to as a colony that was grown on LJ media and found to be AFB-positive after staining with Ziehl Neelsen staining, whereas a strain is an isolate (s) with specific spoligotype pattern. Thus, a strain can consist of a single isolate or several isolates. A strain with a single isolate is termed as a unique strain while a strain with more than one isolate is considered as clustered strain. An online tool Run TB-Lineage http://tbinsight.cs.rpi.edu/run_tb_lineage.html was also used to predict the major lineages using a conformal Bayesian network (CBN) analysis and sub lineage using knowledge based Bayesian network (KBBN).

### Statistical analysis

The statistical analysis was performed using STATA software version 12. Descriptive statistics were used to depict the demographic variables. Pearson chi-square was used to evaluate the association between source site of the isolate and clustering status of the isolates. Similarly, Fisher’s exact test was used to test of sourcesite is significantly associated with type of major lineage identified as well as with the type of dominant strain. Results were considered statistically significant whenever *p*-value was less than 5%.

## Result

### Demographic characteristics of the study participants

Among 338 smear positive sputa samples297 (87.9%) samples were confirmed as culture positive. A total of 297*M. tuberculosis* isolates were utilized to carry out RD9-PCRand spoligotyping analysis of which 281 gave valid spolygotyping data while the remaining 16 isolates did not give any pattern up on spolygotyping. One hundred thirty (46.2%) of 281 isolates obtained from female while 151 (53.7%) were isolated from males. Classification of the study participated on the basis of age showed that 99 (35.3%) were between 18 and 28 years of age, majority (47.7%; 134/281) were originated from Woliso and its surroundings (Table 1).

**Table 1:** Background characteristics tuberculosis patients from whom the *M. tuberculosis* isolates were obtained

Background characteristics	Number (%) of patients
Sex	
Male	151(53.7)
Female	130(46.3)
Age, Years	
18–28	99(35.2)
29–39	72(25.8)
40–60	74(26.3)
> 50	36(12.8)
History of anti-tuberculosis treatment	
Previously treated	10(3.6)
Not previously treated	271(96.4)
Region	
Woliso	134(47.7)
Fiche	97(34.5)
Atat	50(17.8)

Region of difference (RD) 9-based polymerase chain reaction (PCR). The 281 isolates were analyzed using RD9 PCR and the result indicated that all isolates had intact RD9 implying that all the isolates were M. tuberculosis (Fig. 1).

### Spolygotyping result

Spoligotyping of 281 isolates yielded 90 different spolygotype patterns. Of these, 32 strains were clustered containing of 223 (79.3%) isolates and 58 (20.6%) were unique strains (Tables 2 and 4). The overall diversity of the isolates was 32.4%. Out of the 90 spolygtype pattern, 45 were registered in the international data base (Table 2) and the remaining 45 were not found in the database (Table 3). The dominantly identified strains were SIT53, SIT149, and SIT 54 consisting of 43 isolates, 37 isolates, 34 isolates, respectively (Table 2). Classification of the spoligotype patterns using TB-insight RUN TB-Lineage showed that 86.8, 6.4, 5.3, 1.4% of the isolates belonged to the Euro-American lineage, East-African-Indian, Indo-oceanic and *M. africanum,* respectively (Table 2).

**Table 2:**
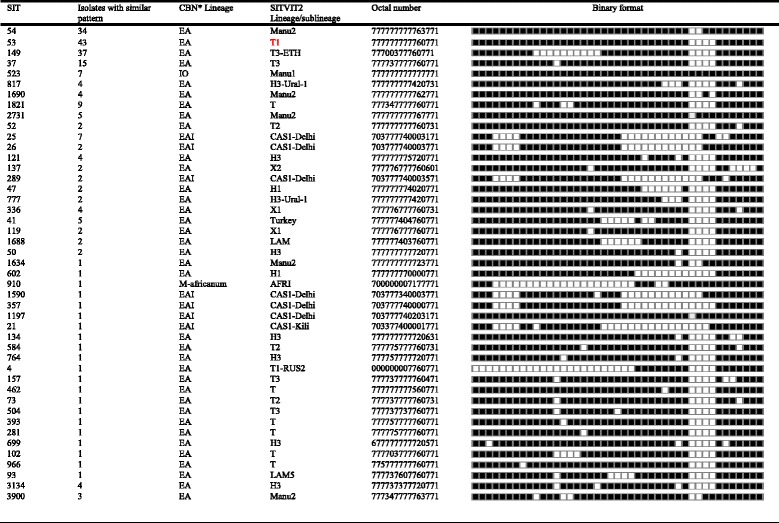
Description of 45 shared-types (SITs;*n* = 224 isolates) which have already been registered in the SITVIT2 or SpolDB4 database and corresponding spoligotyping defined lineages/sublineages starting from a total of 281 *M. tuberculosis* strains isolated in central Ethiopia

A total of 281 isolates grouped into 90 different spolygotype patterns. Of the 90 spolygtype pattern, 45 were registered in the international database while 45 of the strains were not found in the database. Of these 90 strains, 32 were clustered strains consisting of 223 (79.3%) isolates while 58 strains were found as single isolate. The dominantly found strains were SIT 53, SIT 149, and SIT 54 consisting of 43, 37 and 34 isolates, respectively. Classification of the spoligotype patterns using TB-insight RUN TB-Lineage showed that 86.8%, 6.4%, 5.3%, 1.4% of the isolates belonged to the Euro-American lineage, East-African-Indian, Indo-oceanic and *M. africanum*, respectively. *EA* Euro-American; *EAI* East-African Indian; *IO* Indo-Oceanic; *MA* *M. africanum*; *MB* *M. bovis*; *CBN* conformal Bayesian network; *KBBN* knowledge based Bayesian network

**Table 3:**
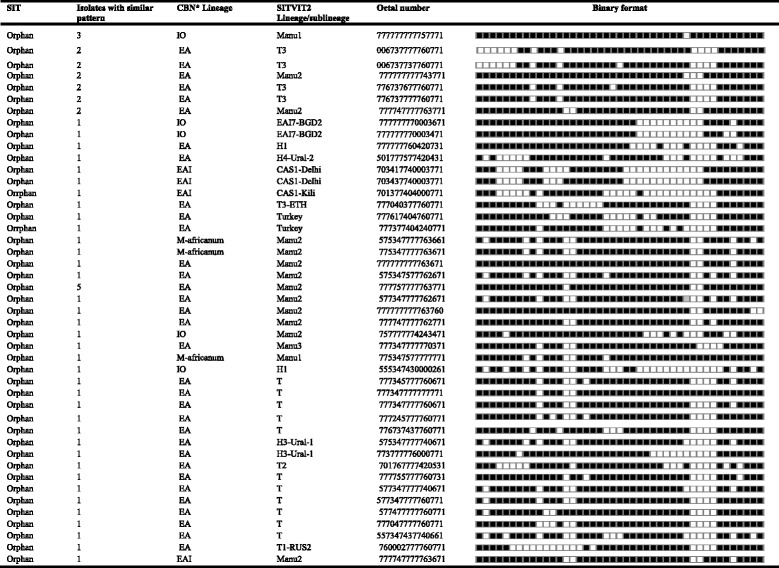
Description of 45 orphan strains (*N* = 57) and corresponding spoligotyping defined lineages/sublineages recorded among *M. tuberculosis* strains starting from a total of 281 *M. tuberculosis* isolated in central Ethiopia

Of the 90 spolygtype pattern, 45 patters consisting of 57 isolates did not match with the patterns available in the database (orphans)

*CBN: conformal Bayesian network; *EA* EuroAmerican; *IO* Indo-Oceanic; *EAI* East African-Indian; *MA* *M. africanum*

### Distribution of strains and lineages in the study area

Of the 281 isolates typed, 134(47.7) were originated from Woliso and its surroundings, with clustering rate of 83.6% (112/134), 97(34.5) were originated from Fiche with clustering rate of 72.2% (70/97). The remaining isolates were from Atat town with clustering rate of 14.6% (41/281). This finding did not show statistically significant difference in the proportion of clustering across the three source sites of the isolates (*p*-value = 0.163). Similarly, there was not significant association of the source site of the isolate with major lineage identified by CBBN (*p*-value = 0.877) as well as the type of dominant isolate (*p*-value = 0.109). The proportions of occurrence of the dominant lineage (Euro-American) at Woliso, Fitche and Atat towns were 85.5% (112/131), 89.1%(90/101) and 85.7% (42/49), respectively (Table 4).

**Table 4:** Distribution of strains and clustering rate in the Study Area (*N* = 281)

Characteristics of the isolates	Number(%) of isolates in the study sites			*P*-value
	Weliso	Atat	Fiche	
Total spolygotyped isolates	134 (47.7)	50 (17.8)	97 (34.5)	
Clustered isolates versus single				
Clustered	112	41	70	0.163
Single	22	9	27	
Clustering rate	83.6%	82%	72.2%	
Major lineage by CBBN				
EA	115	43	86	0.877
EAI	10	4	4	
IO	7	2	6	
MA	2	1	1	
The three dominant Strains				
SIT 53	19	6	18	0.109
SIT 149	17	9	11	
SIT 54	21	8	5	
Orphan strains	21	10	26	

*EA* Euro-American, *EAI* East-African Indian, *IO* Indo-Oceanic, *MA M. africanum*, *MB Mbovis*, *CBN* conformal Bayesian network

## Discussion

In the present study, 281 pulmonary TB cases were recruited from three towns and their surroundings in central Ethiopia for the isolation and identification of the species and strains of MTC causing pulmonary TB in the central Ethiopia. The isolation of was made from the sputum of patients on LJ medium while RD9-based PCR and spoligotyping used for identification of the isolates at the species and strain levels, respectively. An online tool Run TB-Lineage http://tbinsight.cs.rpi.edu/run_tb_lineage.html was also used for grouping lineages using a conformal Bayesian network (CBN) analysis and sub lineage using knowledge based Bayesian network (KBBN).

Out of the 90 different types of spolygotype patterns, 45 of the patterns which consisting majority of the isolates, matched with the patterns registered in the SITVIT2 database while the remaining 45 patterns did not match with the patterns registered in the SITVIT2 or SpolDB4 database. Similar studies that have been conducted in Ethiopia, and thus all the geographic regions of the country have not been covered and as a result all the circulating strains of *M. tuberculosis* have not yet been registered to the SITVIT2 database. Over 75% of the isolates were clustered strains with varies sizes of clustering while about a quarter of the isolates were found as unique strains. There are various assumptions with regard to such findings. Although spoligotyping has less discriminatory power in classifying strains, the finding of many isolates clustering in the same pattern could suggest the presence an on-going transmission of *M. tuberculosis* infection in the specific geographic region. On the other hand, the isolation of many unique strains could suggest the introduction of new strains into that specific region and these strains did not spread in that specific geographic region.

Similar to this study, SIT54 was dominantly isolated by earlier studies conducted in the Addis Ababa City (17), central Ethiopia (18) and in eastern central Ethiopia (19). This strain has been mainly reported to the SITVIT2 and SpolDB4 databasefrom South and East Asia, Middle East including Egypt and USA [10, 18]. Another interesting finding in the study was, similar to other studies conducted in Ethiopia earlier [11, 13] the ancestor strain SIT 523 was found consisting of good numbers of isolates. SIT 523 is characterized by the presence of all 43 spacers and is the ancestor strain of *M. tuberculosis*. It is less likely that strains keep all the 43 spacers intact for a long duration of time since they have to adapt to different pressures through changing their genetic makeup, and hence it is likely that the presence of 43 spacers intact could also be due to mixed infection, which needs further investigation using more powerful molecular techniques.

The *M. tuberculosis* isolated by the present study were belonged to four major lineages including the Euro-American, East-African-Indian, Indo-oceanic and the *M. africanum*. The dominant Lineage wasEuro- American Lineage consisting 86.8% of the isolates. This finding is in agreement with the earlier studies [11, 12, 19] conducted in different regions of Ethiopia. The 2^nd^ and 3^rd^ lineages under which the isolates grouped were East-African-Indian and Indo-oceanic lineage, respectively. Four isolates belonged to *M. africanum* Lineage. *M. africanum* has been reported to be an important cause of human TB in the west African countries including Guinea- Bissau [20], the Gambia [21], Sierra Leone [22], Senegal [23], Burkina Faso [24], Cameroon [25], Nigeria [26], and CôteD’Ivoire [27].

The limitation of this study is the use of only spoligotyping for typing of the isolates. Since the discriminatory power of spoligotyping is low, the finding of this study could lead to an overestimation of clustering because of the failure to differentiate between recent transmissions and mixed infections. In contrast to spoligotyping, MIRU-VNTR or WGS genotyping allow for a high-resolution and discrimination of the isolates for epidemiological studies and a valid phylogenetic strain classification [28–30].

## Conclusion

The identification of clustered and new strains using spolygotyping in present study could not give conclusive report, as spoligotyping has low discriminatory power. Hence, further characterization of these isolates using MIRU-VNTR or WGS was recommended.

## Abbreviations

CBN: Conformal bayesian network
CI: Confidence intervals
DNA: Deoxo-nuclic acid
DR: Direct repeats
EA: Euro-American
EAI: East-African -indian
IO: Indo-occenic
IS: Insertion sequence
KBBN: Knowledge based bayesian network
LJ: Löwenstein —jensen medium
*M. tb*: *Mycobacterium tuberculosis*
MA: M.-africanum
MDR-TB: Multi drug resistance tuberculosis
MIRU-VNTR: Mycobacterial interspersed repetitive unit-variable number tandem repeat
MTBC: *Mycobacterium tuberculosis* complex
PCR: Polymerase chain reaction
RD: Regions of difference
SIT: Shared international type
SNPs: Single nucleotide polymorphisms
TB: Tuberculosis
WGS: Whole genome sequence
WHO: World Health Organization
